# *Spiranthes hachijoensis* (Orchidaceae), a new species within the *S. sinensis* species complex in Japan, based on morphological, phylogenetic, and ecological evidence

**DOI:** 10.1007/s10265-023-01448-6

**Published:** 2023-03-17

**Authors:** Kenji Suetsugu, Shun K. Hirota, Hiroshi Hayakawa, Shohei Fujimori, Masayuki Ishibashi, Tian-Chuan Hsu, Yoshihisa Suyama

**Affiliations:** 1 Department of Biology, Graduate School of Science, Kobe University, Kobe, Hyogo 657-8501 Japan; 2 Institute for Advanced Research, Kobe University, 1-1 Rokkodai, Nada-Ku, Kobe, Hyogo 657-8501 Japan; 3 Field Science Center, Graduate School of Agricultural Science, Tohoku University, 232-3 Yomogida, Naruko-Onsen, Osaki, Miyagi 989-6711 Japan; 4 Botanical Gardens, Osaka Metropolitan University, 2000 Kisaichi, Katano City, Osaka, 576-0004 Japan; 5 Museum of Natural and Environmental History, Shizuoka, 5762 Oya, Suruga, Shizuoka, Shizuoka Japan; 6 Graduate School of Life and Environmental Sciences, University of Tsukuba, 1-1-1 Tennodai, Tsukuba, Ibaraki 305-8572 Japan; 7 Tokorozawa City, Saitama 359-0024 Japan; 8 Botanical Garden Division, Taiwan Forestry Research Institute, No. 53, Nanhai Rd, Taipei, 100 Taiwan

**Keywords:** Integrative taxonomy, Orchidaceae, Reproductive isolation, SNP data, Speciation, Species delimitation, *Spiranthes sinensis* species complex

## Abstract

The systematics of the Old World *Spiranthes sinensis* (Pers.) Ames species complex (Orchidaceae) has been complicated by its wide distribution and morphological variations. Within the species complex, *S. australis* Lindl. has been generally accepted as the only *Spiranthes* Rich. species distributed on the Japanese mainland. The present study provides morphological, phylogenetic, and ecological evidence for the recognition of *S. hachijoensis* Suetsugu as a new species of the *S. sinensis* species complex on the Japanese mainland. *Spiranthes hachijoensis* is morphologically similar to *S. hongkongensis* S.Y. Hu & Barretto and *S*. *nivea* T.P. Lin & W.M. Lin, sharing a degenerated rostellum, pollinia without a viscidium, and distinctly trilobed stigma. However, the taxon can be morphologically distinguished from *S. hongkongensis* by its glabrous rachis, ovaries, and sepals, and from *S*. *nivea* by its papillate labellum disc, larger papillate basal labellum callosities, and glabrous rachis, ovaries, and sepals. The autogamy and flowering phenology (i.e., earlier flowering) of *S. hachijoensis* are most likely responsible for premating isolation from the sympatric *S. australis*. A MIG-seq-based high-throughput molecular analysis indicated that the genetic difference between *S. hachijoensis* and its putative sister species *S. sinensis* is comparable to, or even greater than, the genetic difference between pairs of other species within the *S. sinensis* species complex. Our multifaceted approach strongly supports the recognition of *S. hachijoensis* as a morphologically, phenologically, phylogenetically, and ecologically distinct species.

**Supplementary Information:**

The online version contains supplementary material available at 10.1007/s10265-023-01448-6.

## Introduction

The genus *Spiranthes* Rich. (Orchidaceae) includes approximately 50 species that are widely distributed across the tropical and temperate regions of the Americas, Eurasia, and Australia (Dueck et al. 2014; Pace et al. 2019; Surveswaran et al. 2017, 2018). Nonetheless, the delimitation of closely related *Spiranthes* species based on morphology alone is hindered by phenotypic plasticity, convergent morphological features, and hybridization (Dueck et al. 2014; Pace and Cameron 2017; Pace et al. 2019; Suetsugu et al. 2020; Surveswaran et al. 2017, 2018; Tao et al. 2018). In particular, the systematics of the Old World *S. sinensis* (Pers.) Ames species complex has been complicated by the wide distribution and morphological variation of the group (Hsu and Chung 2014; Hu and Barretto 1976; Lin and Lin 2011).

Recently, the systematics of the *S. sinensis* species complex has partially been resolved using molecular approaches (Pace et al. 2019; Surveswaran et al. 2017, 2018). Working under an integrative phylogenetic species concept, several recent studies have performed extensive molecular phylogenetic sampling of the *S*. *sinensis* species complex (Pace and Cameron 2017; Surveswaran et al. 2017, 2018). The combination of this molecular evidence with phenological and morphological data has facilitated the recognition of seven distinct taxa within the complex (Pace et al. 2019; Surveswaran et al. 2018). However, although the recognition of seven distinct taxa within the complex is plausible,, there is some controversy over the most appropriate scientific names for the seven taxa due to their complex morphological features (Pace and Cameron 2020; Surveswaran et al. 2020). Here, we basically followed Pace et al. (2017), with slight modification as detailed by Suetsugu and Hsu (2023), accepting the following seven species (*S*. *australis* Lindl., *S*. *flexuosa* Lindl., *S*. *hongkongensis* S.Y. Hu & Barretto, *S*. *maokensis* M.C. Pace, *S*. *nivea* T.P. Lin & W.M. Lin [replacing *S. suishaensis* in Pace et al. (2017)], *S*. *sinensis*, and *S*. *sunii* Boufford & Wen H. Zhang) within the *S. sinensis* species complex. Nonetheless, given that phenological divergence could have facilitated speciation without other observable or measurably significant morphological differences (other than pubescence; Pace et al. 2019) and that some taxa within the *S. sinensis* complex have recently been recognized (Pace et al. 2019; Surveswaran et al. 2017), it is likely that more species remain unrecognized and misidentified within widespread taxa such as *S. australis* and *S. sinensis*.

Three species (*S. australis*, *S. sinensis*, and *S. hongkongensis*) have been recognized in Japan. It should be noted that, although Pace et al. (2019) reported *S. flexuosa* from Japan, this was likely the result of an incorrect assignment, as discussed previously (Surveswaran et al. 2020). Our intensive herbarium and field surveys failed to provide evidence of the occurrence of *S. flexuosa* in Japan. *Spiranthes sinensis* can be characterized by its phenology (spring flowering) and glabrous rachis and ovaries (Hayakawa et al. 2013; Pace et al. 2019; Suetsugu and Hayakawa 2016). Meanwhile, *S*. *australis* can be characterized by its early summer or autumn flowering phenology and glandular pubescent rachis, stems, and ovaries (Hayakawa et al. 2013; Pace et al. 2019; Suetsugu and Hayakawa 2016; Tsukaya 1994, 2005). Finally, *S. hongkongensis*, with glandular pubescent rachis, floral bracts, ovaries, and sepals, can be distinguished from both *S. sinensis* and *S. australis* by its degenerated rostellum, pollinia without a viscidium, shorter column, and distinctly trilobed stigma (Hsu and Chung 2014; Hu and Barretto 1976; Suetsugu and Hayakawa 2019). These species also show some geographic segregation, at least in Japan and Taiwan. *Spiranthes australis* is distributed on mainland Japan (northern Ryukyu Islands and northward), whereas *S. sinensis* and *S. hongkongensis* are restricted in the more southern areas (central and southern Ryukyu Islands and Taiwan [*S. sinensis*] and Ishigaki Island, southern Ryukyu, and Taiwan [*S. hongkongensis*]) (Maekawa 1971; Satomi 1982; Suetsugu and Hayakawa 2019). Although glabrous *Spiranthes* individuals have been reported from mainland Japan on rare occasions, molecular analyses have suggested that most of these individuals are variants of *S. australis*, rather than a range extension of *S. sinensis* (Hayakawa et al. 2013; Suetsugu and Hayakawa 2016).

During extensive field surveys focusing on Japanese *Spiranthes* individuals for phylogeographic studies (Suetsugu, unpublished data), multiple populations of the unknown *Spiranthe*s taxon with glabrous rachis and ovaries have been found on mainland Japan. Interestingly, the unknown taxon often co-occurs with *S. australis* but flowers approximately 1 month earlier, thereby isolating the taxa reproductively. Given that the features “pubescence” and “flowering time” are often used as diagnostic traits in *Spiranthes* (Hayakawa et al. 2013; Pace et al. 2019; Suetsugu and Hayakawa 2016), the glabrous individuals may reflect a range extension of *S. sinensis.* Meanwhile, the geographical separation of *S. sinensis* and the unknown taxon suggests that the glabrous individuals are variants of *S. australis* (Maekawa 1971; Hayakawa et al. 2013; Satomi 1982; Suetsugu and Hayakawa 2016). However, additional morphological curiosities, such as a weakly open flower and a weakly recurved labellum (usually recurved by < 270°), which are not typical variations of either *S. sinensis* or *S. australis* (Surveswaran et al. 2017), suggest that the individuals represent an overlooked species.

Accordingly, we have used an integrative taxonomic approach to determine whether the unknown taxon represents a distinct entity within the *S. sinensis* complex. Species delimitation that explicitly considers ecological and phylogenetic differences has critically advanced the current understanding and evaluation of biodiversity (Barrett and Freudenstein 2011). Over the last two decades, these integrative taxonomic approaches have provided more robust estimates of biodiversity among taxonomically challenging species (Barrett and Freudenstein 2011; Barrett et al. 2022; Botes et al. 2020; Pace et al. 2019; Suetsugu et al. 2023). The present study provides morphological, phylogenetic, and ecological evidence that supports the recognition of *S. hachijoensis* Suetsugu, named after its type locality, which sustains the largest population of the taxon.

## Materials and methods

### Morphological observations

Morphological characteristics of the *S. sinensis* species complex were compared using herbarium specimens from CBM, KYO, KPM, TAIF, TI, and TNS and living individuals of *S. australis*, *S. flexuosa*, *S. hachijoensis*, *S. hongkongensis*, *S. nivea* (including *S. nivea* var. *papillata*), and *S. sinensis* collected in Laos, Japan, and Taiwan during fieldwork between 2012 and 2022 (Table S1). Morphological characters were visually observed under a Leica M165C stereomicroscope and measured using a digital caliper. The dissected flowering specimens were photographed using an Olympus OM-D E-M1 Mark II digital camera equipped with an Olympus 30 mm macro lens or a Leica MC170 HD digital camera attached to a Leica M165C stereo microscope. Morphological differences among species of the *S. sinensis* complex, including *S. maokensis* and *S. sunii* which we could not obtain living materials for analysis, were investigated by reviewing relevant literature and online digitized plant collections such as JSTOR Global Plants (http://plants.jstor.org/). At least one voucher specimen for newly collected samples from each population during our field survey was deposited in KYO, TAIF, TNS, RYU, and SPMN (Table S1). The herbarium acronyms follow the Index Herbariorum (Thiers 2022).

### Reproductive biology

The reproductive biology of a *S. hachijoensis* population in Ichihara-shi (Chiba Pref., Japan) was investigated from mid-May to early-June, 2016. Because previous studies suggested that closely related *Spiranthes* species are pollinated by bees (e.g., *Megachile* and *Apis* species; Iwata et al. 2012; Suetsugu and Abe 2021; Tao et al. 2018), floral visitors were surveyed during the daytime (10:00–16:00) for a total of 18 h.

To investigate the breeding system of the species, hand-pollination experiments were performed using five treatments. Because flowers in the upper of the inflorescence often do not bear fruit even when pollinated due to resource limitations, 5 flowers from the base of the inflorescence on each individual were used to accurately determine the effect on pollination. For the autonomous autogamous treatment, flowers were bagged before anthesis using a fine mesh to exclude pollinators (20 flowers from 4 individuals). For the manually autogamous treatment, pollinaria were removed and used to hand-pollinate the same flowers, after which the flowers were bagged with a fine mesh (20 flowers from 4 individuals). For the manually allogamous treatment, pollinaria were removed and used to hand-pollinate flowers on plants at least 1 m away, after which flowers on the recipient plant were bagged with fine mesh (20 flowers from 4 individuals). For the open treatment, flowering plants were randomly tagged and allowed to develop fruit under natural conditions (40 flowers from 8 individuals). The experimental plants were monitored intermittently for fruit development over the following 3 weeks. The proportion of seeds with at least one well-developed embryo was then evaluated by screening 50 randomly selected seeds from each capsule. In addition, because polyembryonic seeds have been associated with agamospermy (vs. monoembryonic seeds with sexual reproduction) in *Spiranthes* (Catling 1982; Sun 1996), the occurrence of agamospermous seed development was investigated by examining the number of embryos in a seed.

Finally, the fruit set among the treatment groups was compared using the Fisher’s exact test. The effects of pollination treatment on seed viability were evaluated using ANOVA.

### MIG-seq-based high-throughput genomic analysis

Eight *S. australis* individuals, twenty-eight *S. hachijoensis* individuals, and six *S. sinensis* individuals collected throughout Japan were used for multiplexed inter-simple sequence repeat genotyping (MIG)-seq analysis. Five *S. sinensis* individuals from Taiwan, four *S. hongkongensis* individuals from China and Taiwan, three *S. nivea* individuals (including two *S. nivea* var. *papillata* individuals) from Taiwan, and a single *S. flexuosa* individual from Laos were included in the comparative study (Table S1). Genomic DNA was extracted from silica-dried leaves using the CTAB method (Doyle and Doyle 1990). A MIG-seq library for the 55 *Spiranthes* samples was prepared as described by Suyama et al. (2022) and sequenced using a MiSeq system (Illumina, San Diego, CA, USA) and MiSeq Reagent Kit v3 (150 cycle; Illumina). The raw MIG-seq data were deposited in the DDBJ Sequence Read Archive (SRA accession number PRJNA907989).

After removing primer sequences and low-quality reads (Suetsugu et al. 2021), 15,943,788 reads (289,887 ± 6998 reads per sample) were obtained from 17,873,310 raw reads (324,969 ± 7906 reads per sample). Stacks 2.60 pipeline was used for de novo single nucleotide polymorphism (SNP) discovery (Rochette et al. 2019), with the following parameters: minimum depth of coverage required to create a stack (*m*) = 3, maximum distance allowed between stacks (*M*) = 2, and number of mismatches allowed between sample loci while building the catalog (*n*) = 2. SNP sites with high heterozygosity (Ho ≥ 0.6) were removed, and SNP sites with fewer than three minor alleles were filtered out. Then, a SNP was excluded if the number of samples shared by the SNP was below the reference value R (the minimum proportion of samples that retained a SNP). We used four conditions referring to the threshold for the minimum number of samples that retained a SNP to determine the robustness of the results. Only SNPs retained by 6 (R = 0.1), 17 (R = 0.3), 28 (R = 0.5), and 44 (R = 0.8) or more samples were included in the datasets. Finally, 13,809 SNPs in 6630 loci, 7972 SNPs in 3438 loci, 5275 SNPs in 1976 loci, and 538 SNPs in 203 loci were included for subsequent analysis.

A SNP-based maximum-likelihood (ML) phylogeny was inferred using RAxML v. 8.2.10 (Stamatakis 2014), with a GTR substitution model with Lewis’ ascertainment bias correction and 1000 bootstrap replicates. Additionally, a Neighbor-Net network was constructed using the uncorrected *p* distance matrix and ignoring ambiguous sites in SplitsTree4 4.14 (Huson and Bryant 2006).

## Results and discussion

### Phylogenetic distinctness of* Spiranthes hachijoensis*

**Fig. 1:**
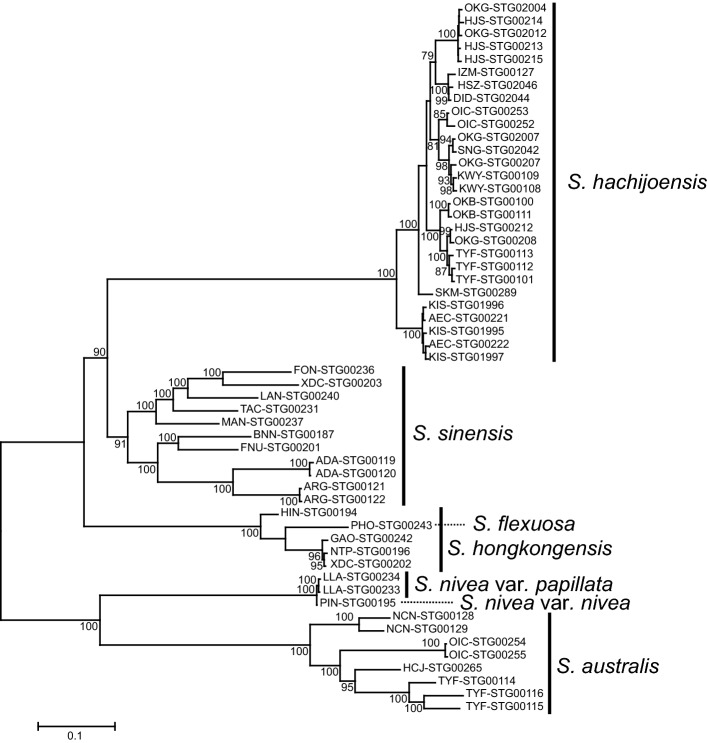
Phylogenetic tree of *Spiranthes hachijoensis* and its closely related taxa reconstructed from MIG-seq data based on 6630 loci 13,809 SNPs (R = 0.1). Nodes supported by bootstrap values < 70% are not shown. Branch length represents the average number of substitutions per site

**Fig. 2:**
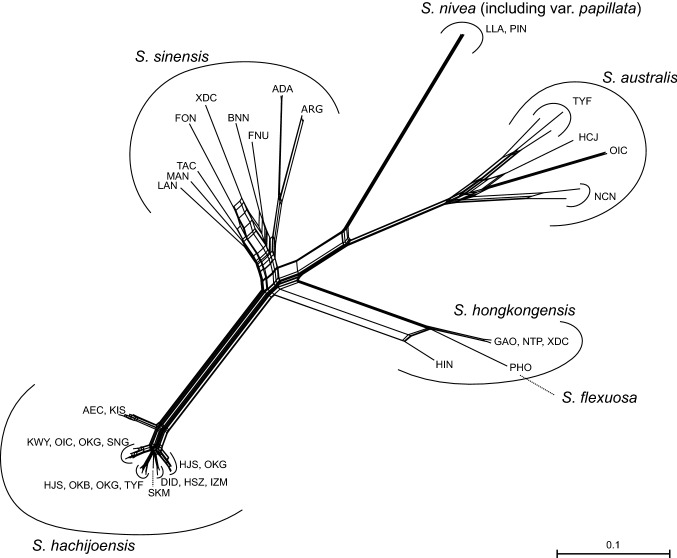
Neighbor-Net network of *Spiranthes hachijoensis* and its closely related taxa reconstructed based on the uncorrected *p* distance calculated from 13,809 SNPs (R = 0.1). Branch length represents the average number of substitutions per site

A ML phylogenetic tree reconstructed from MIG-seq data indicated that *S. hachijoensis* is more closely related to the allopatric *S. sinensis* than to the sympatric *S. australis* (Fig. 1 and Figs. S1, S2)*.* It should be noted that the choice of different values for the parameter R resulted in some variations in the topology of the phylogenetic tree. The ML phylogenetic tree using R = 0.1 suggested that *S. hachijoensis* forms a relatively well-supported clade with *S. sinensis* (Fig. 1), while the phylogenetic tree using R = 0.5 or R = 0.8 suggested that *S. hachijoensis* forms a clade with *S. hongkongensis* + *S. flexuosa*, despite weak bootstrap support (Fig. S1). Regardless of the discrepancy among phylogenetic trees reconstructed using different numbers of loci, all the phylogenetic analyses indicated that the genetic difference between *S. hachijoensis* and *S. sinensis* is similar to, or even greater than, the genetic differences observed between pairs of other species within the complex (Figs. 1, 2 and Figs. S1, S2). Therefore, all molecular data support the recognition of *S. hachijoensis* as an independent species.

Although *S. hachijoensis* is morphologically most similar to *S*. *hongkongensis*, the ML and Neighbor-Net phylogenetic analyses also indicated that *S. hachijoensis* represents a distinct genetic cluster from *S*. *hongkongensis* (Figs. 1, 2 and Figs. S1, S2). The molecular results are consistent with our preliminary karyological study showing 2*n* = 30 for *S. hachijoensis* (K. Suetsugu & N. Nakato, unpublished data). In contrast, the chromosome counts, analysis of isozyme loci, and molecular phylogenetics indicated that *S*. *hongkongensis* is an allotetraploid (2*n* = 60) resulting from a hybridization event between *S*. *sinensis* and *S*. *flexuosa* (Pace et al. 2019; Sun 1996; Surveswaran et al. 2018). Furthermore, even in the localities where *S. hachijoensis* and *S. australis* grow sympatrically (Toyofusa-shi, Chiba Pref. [TYF], Okago, Hachijo Island, Tokyo Metropolis [OKG], and Ooi-cho, Ena-shi, Gifu Pref. [OIC]), the genetic composition of both species did not differ significantly from that of species in the other localities, and no individuals possessing genetic components of the other species were detected (Figs. 1, 2).

**Fig. 3:**
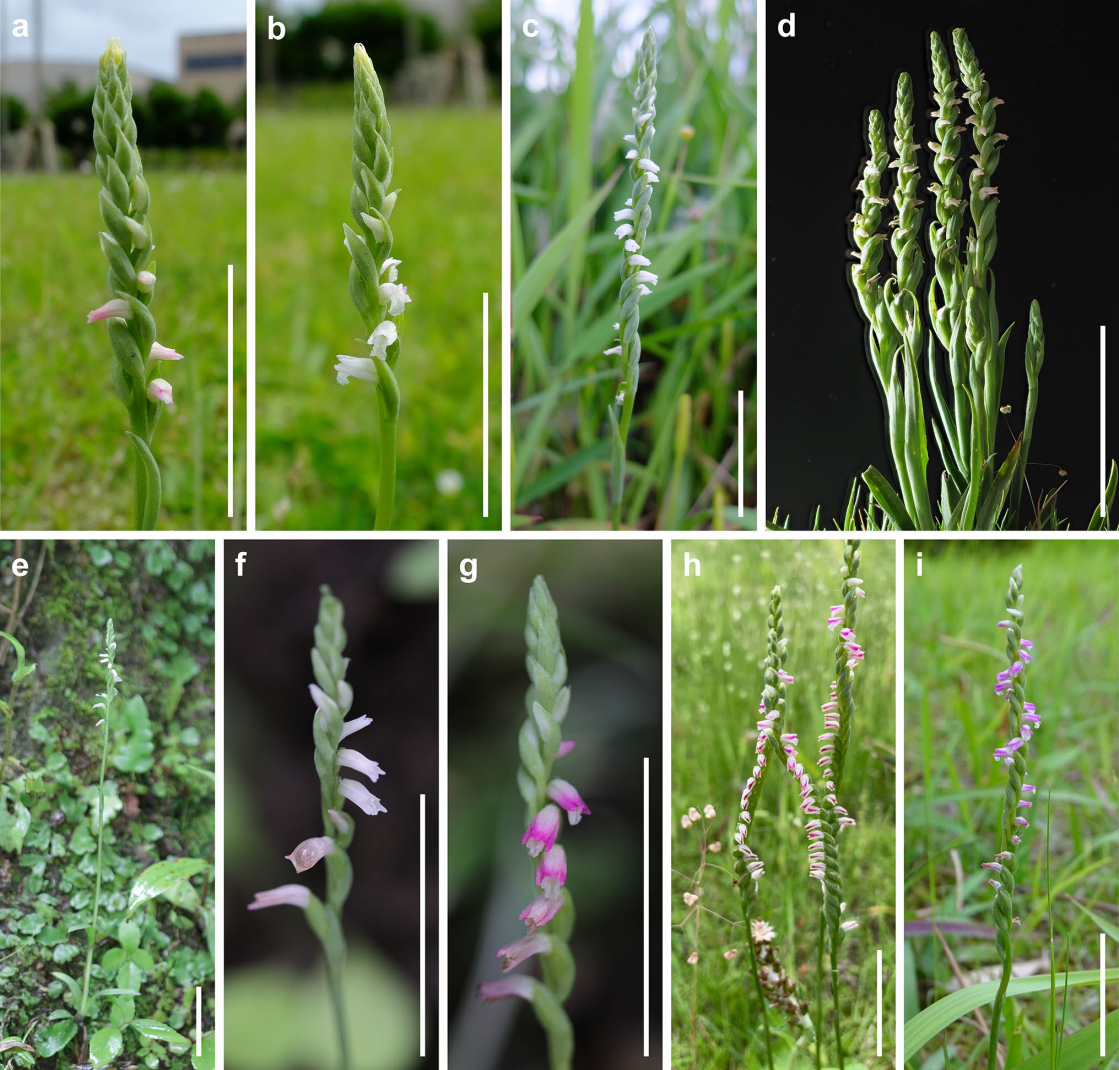
*Spiranthes hachijoensis* in its natural habitat in Japan. **a**–**d** Flowering individual observed on Hachijo Island. **e**, **f** Flowering individual observed in Ichihara-shi, Chiba Pref. **g** Flowering individual observed in Kimitsu-shi, Chiba Pref. **h** Flowering individual observed in Nagoya-shi, Aichi Pref. **i** Flowering individual observed in Kagoshima-shi, Kagoshima Pref. Scale bars: 30 mm

Remarkably, even though *S. hachijoensis* is somewhat polymorphic in flower coloration and floral bract length (Fig. 3), the species showed relatively weak genetic variation within and between populations (Figs. 1, 2 and Figs. S1, S2), which may be due to its predominantly autogamous breeding system. Both the ML and Neighbor-Net phylogenetic analyses also indicated that *S. nivea* var. *nivea* and *S. nivea* var. *papillata* show little genetic differentiation (Figs. 1, 2 and Figs. S1, S2). The phylogenetic data supported *S. nivea* var. *papillate* as an intraspecific taxon, despite the morphological distinctness (Hsu and Chung 2016). Overall, the present study indicates that MIG-seq data can provide valuable insight into species delimitation within morphologically ambiguous groups.

### Ecological distinctness of *Spiranthes hachijoensis*

The pollination experiments revealed that *S. hachijoensis* consistently achieves a high fruit set, even in the absence of pollinators. Therefore, *S. hachijoensis* reproduction is not pollinator-limited under natural conditions. In addition, the proportion of seeds having a well-developed embryo was not significantly affected by pollination treatment (Table 1). All the examined seeds were monoembryonic or lacked embryos, which suggested that the high fruit set of the species was due to autogamy rather than agamospermy. Further investigation revealed that degenerated rostellum tissue, which allows contact between pollen masses and the upper portion of the stigmatic surface, was the most likely mechanism responsible for autonomous self-pollination in this species.

**Table 1:** Effects of pollination treatment on fruit set, seed mass and proportion of seeds having an embryo in *Spiranthes hachijoensis*

	Agamospermy	Autonomous autogamy	Manual autogamy	Manual allogamy	Open
Fruit set (%)	0^a^	95.0^b^	100.0^b^	95.0^b^	97.5^b^
Seeds with embryo (%)	0^a^	81.6 ± 10.8^b^	83.8 ± 9.6^b^	83.3 ± 9.9^b^	83.9 ± 8.9^b^

Different superscript letters indicate significant differences (*P* < 0.05) between treatments. The proportion of seeds having an embryo are expressed by mean ± SD

During floral visitor surveys, syrphid flies (*Sphaerophoria* sp.) and sweat bees (*Lasioglossum* sp.) were observed landing and spending time on *S. hachijoensis* flowers but not entering the inner part of the flowers. Consequently, no pollen grains were introduced to the flowers by the visitors, and the pollinia remained intact within the anther. Thus, no effective pollinators were documented. Notably, *S. hachijoensis* does not have a viscidium; therefore, its pollinaria exhibit almost no adhesive properties, thereby limiting the attachment of pollinaria to potential pollinators. This provides further evidence that autogamy is the dominant (if not exclusive) reproductive strategy of this species*.* Furthermore, because autogamy has been recognized as a mechanism of reproductive isolation between sympatric outcrossing and autogamous species (Suetsugu 2022; Sun 1996), the predominantly autogamous breeding system of *S. hachijoensis* likely facilitates the reproductive isolation of the species from the sympatric and outcrossing species *S. australis.*

*Spiranthes hachijoensis* and *S. australis* are also isolated by their phenology*.* Such phenological isolation has substantial potential to facilitate reproductive isolation, since asynchrony in flowering time can reduce heterospecific pollen deposition, thereby promoting conspecific mating (Lowry et al. 2008; Pace et al. 2019; Sun 1996). In warm-temperate regions of Japan, *S. australis* blooms from mid-June to early-July, i.e., approximately 1 month after *S. hachijoensis,* which flowers from early-May to early-June. Therefore, it is likely that both the autogamy and early flowering phenology of *S. hachijoensis* contribute to the premating isolation and are responsible for the absence of natural hybrids between *S. hachijoensis* and *S. australis*, even where the two species co-occur. Notably, *S. hachijoensis* and its putative outcrossing ancestral species *S. sinensis* are also isolated by their disjunct distribution and differential flowering phenology*. Spiranthes hachijoensis* is distributed at latitudes higher than the Ryukyu Islands, whereas *S. sinensis* is restricted to the more southern areas (whose northern limit is Amami-Ohshima Island, Ryukyu Islands) (Maekawa 1971; Satomi 1982; Suetsugu and Hayakawa 2019). Moreover, *S. sinensis* blooms in Japan from late-February to mid-April, i.e., at least 1 month earlier than *S. hachijoensis.* Accordingly, based on the biological species concept, which defines a species as members of populations that interbreed in nature, *S. hachijoensis* is also distinct from *S. sinensis*, due to geographical, flowering phenological, and reproductive isolation*.*

Pace et al. (2019) reported that the component taxa of the *S. sinensis* species complex exhibit varying degrees of autogamy and suggested that autogamy has contributed to intraspecific morphological variability and, in some instances, speciation. For example, flowers of *S. australis* in New Zealand (also known as *S. novae-zelandiae* Hook. f.), which lack a rostellum and viscidium, form a weakly supported phylogenetic subclade within *S. australis* (Frericks et al. 2018; Pace et al. 2019). However, since similar levels of molecular variation have been documented in other *Spiranthes* species without morphological divergence (Pace and Cameron 2017), the recognition of *S. novae-zelandiae* as a distinct taxon has not yet been warranted (Pace et al. 2019). In contrast, the autogamous species *S. nivea* and *S. hongkongensis* are usually treated as distinct taxa because they exhibit distinct molecular and morphological characteristics (Pace et al. 2019; Surveswaran et al. 2018). Therefore, given not only reproductive isolation (for the biological species concept) but also genetic isolation (for the phylogenetic species concept) and morphological distinction (for the morphological species concept; see later section), we consider *S. hachijoensis* to be a separate species.

### Morphological distinctness of *Spiranthes hachijoensis*

**Fig. 4:**
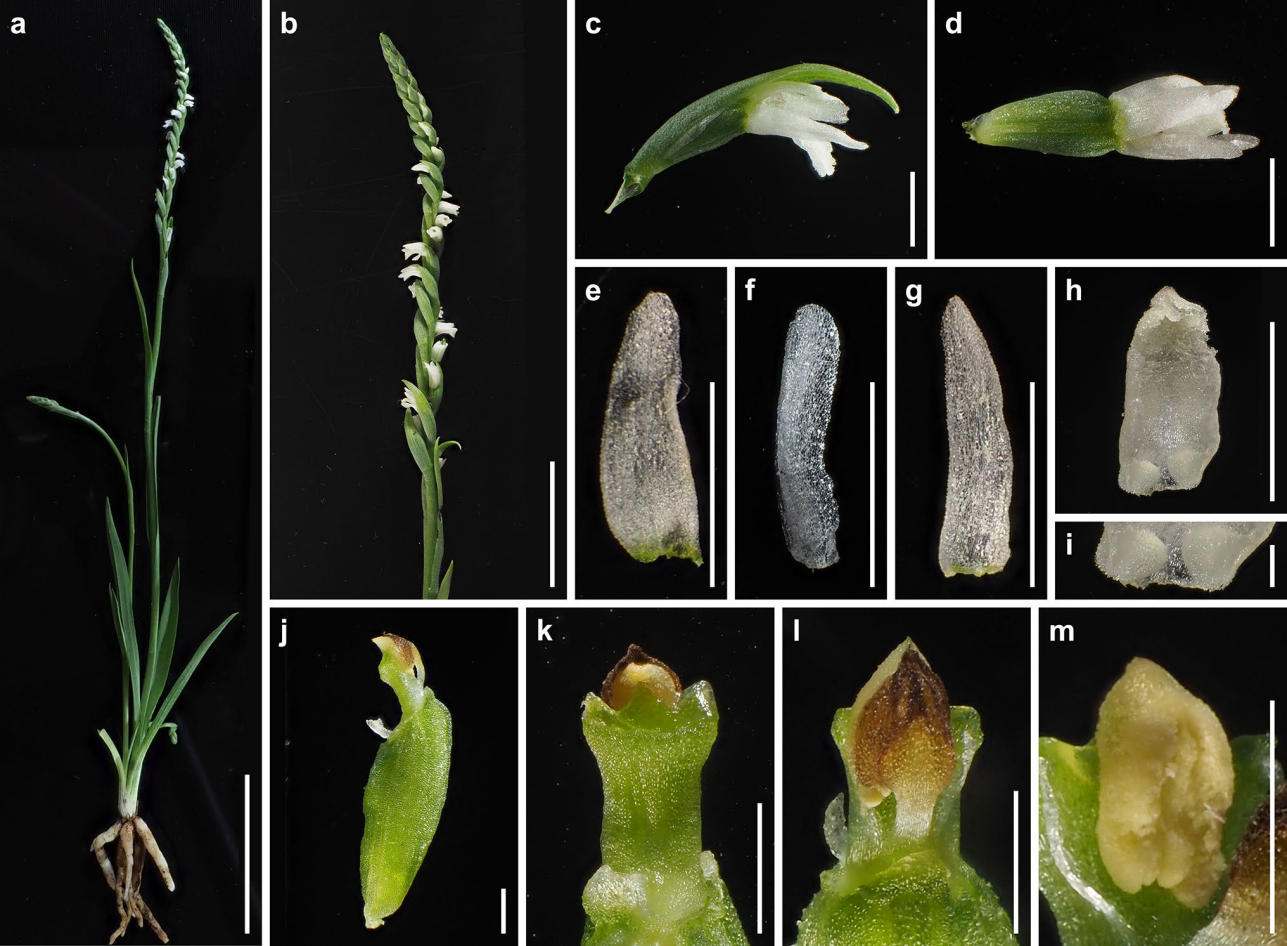
*Spiranthes hachijoensis* (*Kyoko Kaneda & Mayumi Sugiura Ss205-1*, KYO) on Hachijo Island, Tokyo Pref., Japan. **a** Habit. **b** Inflorescence. **c** Flower, lateral view. **d** Flower, top view. **e** Dorsal sepal. **f** Petal. **g** Lateral sepal. **h** Labellum. **i** Close-up of basal labellum callosities. **j** Ovary and column. **k** Column, bottom view. **l** Column, top view. **m** Pollinia without a viscidium. Scale bars: **a** 50 mm, **b** 20 mm, **c**–**h** 3 mm, **i** 0.5 mm, and **j**–**m** 1 mm

**Fig. 5:**
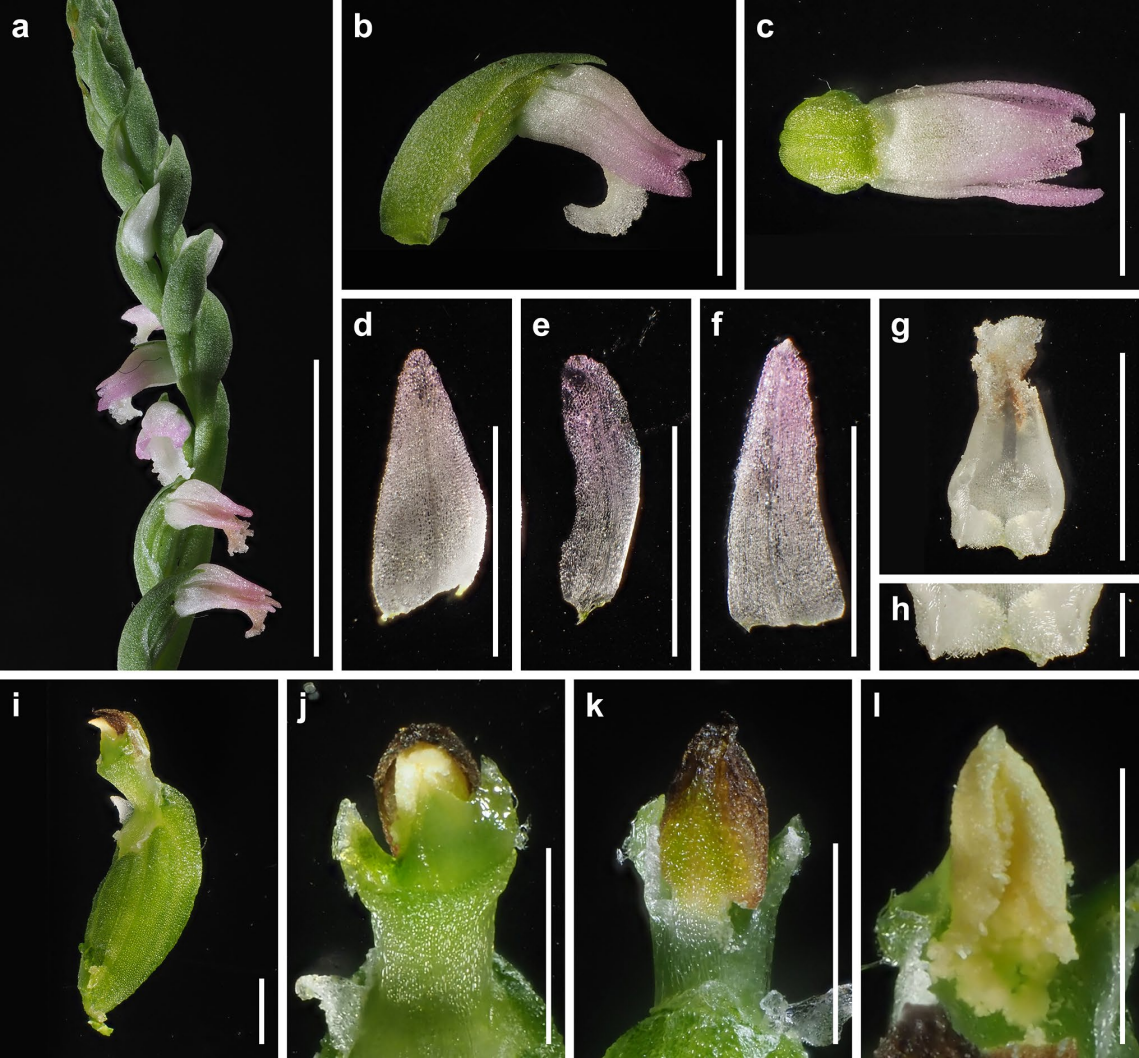
*Spiranthes hachijoensis* (*Kyoko Kaneda & Mayumi Sugiura Ss205-4*, KYO) on Hachijo Island, Tokyo Pref., Japan. **a** Inflorescence. **b** Flower, lateral view. **c** Flower, top view. **d** Dorsal sepal. **e** Petal. **f** Lateral sepal. **g** Labellum. **h** Close-up of basal labellum callosities. **i** Ovary and column. **j** Column, bottom view. **k** Column, top view. **l** Pollinia without a viscidium. Scale bars: **a** 20 mm, **b**–**g** 3 mm, **h** 0.5 mm, and **i**–**l** 1 mm

**Fig. 6:**
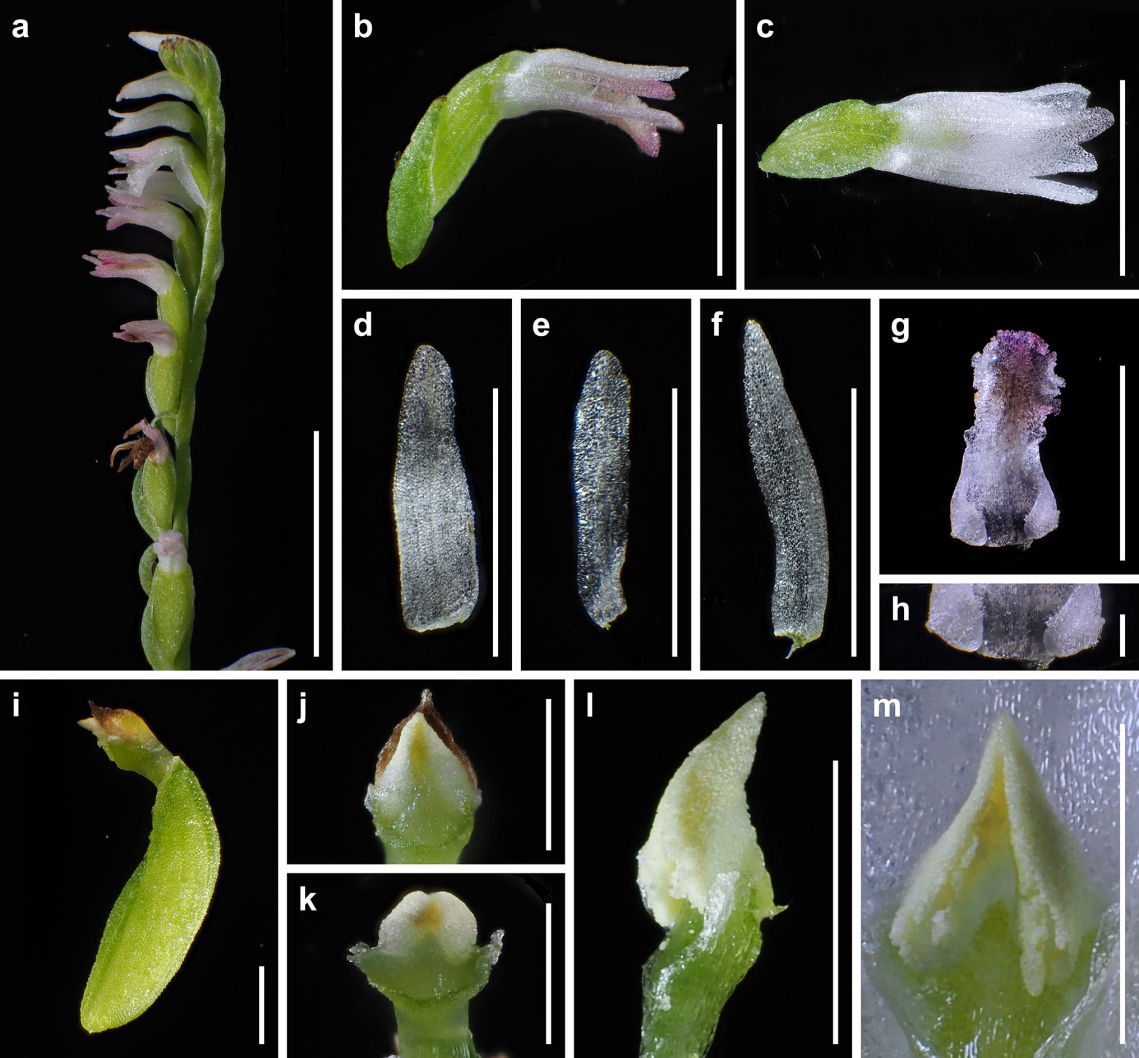
*Spiranthes hachijoensis* (*Emiko Kato KS244*, KYO) in Ichihara-shi, Chiba Pref., Japan. **a** Inflorescence. **b** Flower, lateral view. **c** Flower, top view. **d** Dorsal sepal. **e** Petal. **f** Lateral sepal. **g** Labellum. **h** Close-up of basal labellum callosities. **i** Ovary and column. **j**, **k** Column, bottom view. **l** Column, lateral view. **m** Pollinia without a viscidium. Scale bars: **a** 20 mm, **b**–**g** 3 mm, **h** 0.5 mm, and **i**–**m** 1 mm

**Fig. 7:**
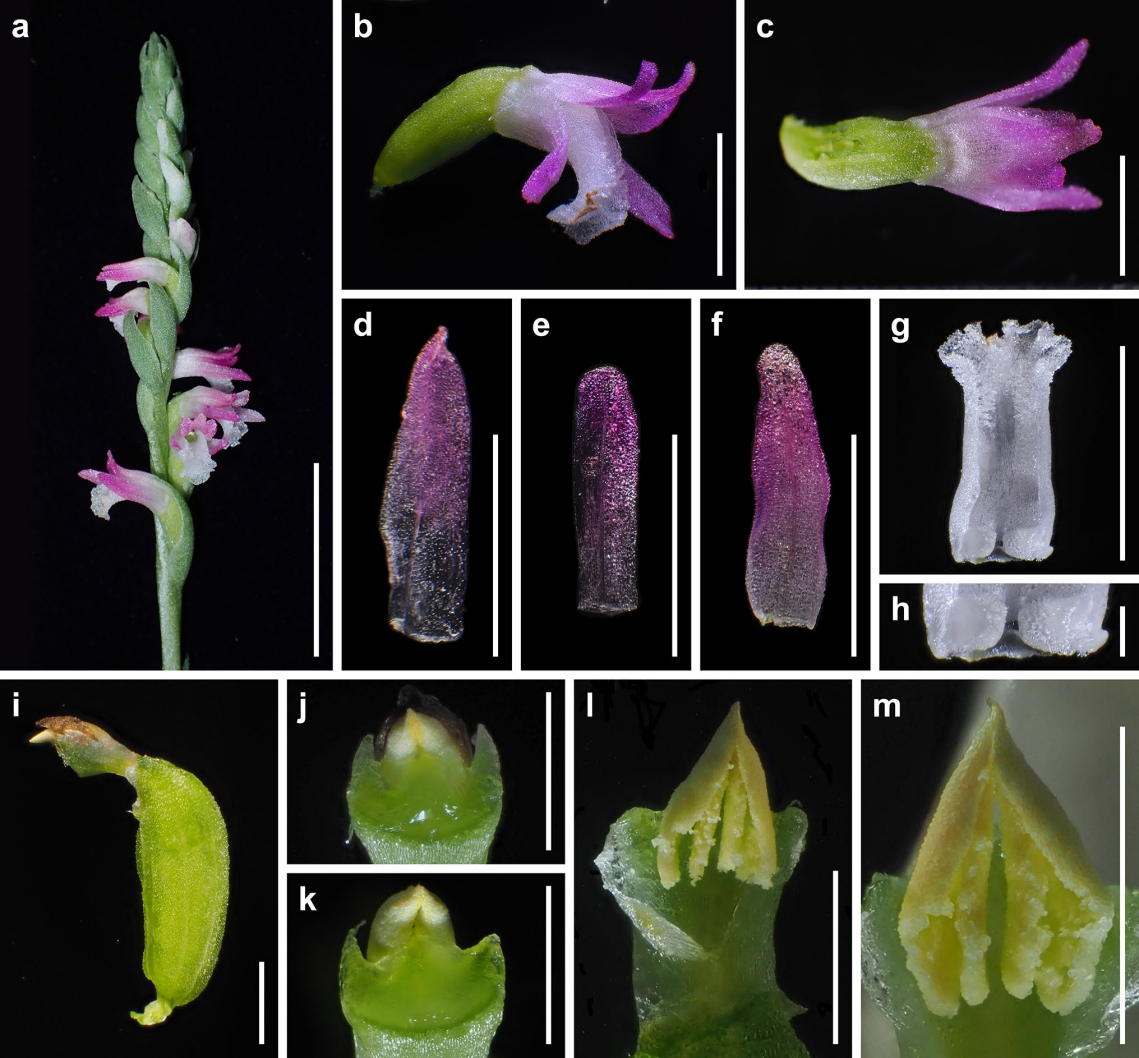
*Spiranthes hachijoensis* (*Katsumi Iwahori KS246*, KYO) in Ena-shi, Gifu Pref., Japan. **a** Inflorescence. **b** Flower, lateral view. **c** Flower, top view. **d** Dorsal sepal. **e** Petal. **f** Lateral sepal. **g** Labellum. **h** Close-up of basal labellum callosities. **i** Ovary and column. **j**, **k** Column, bottom view. **l** Column, top view. **m** Pollinia without a viscidium. Scale bars: **a** 20 mm, **b**–**g** 3 mm, **h** 0.5 mm, and **i**–**m** 1 mm

**Fig. 8:**
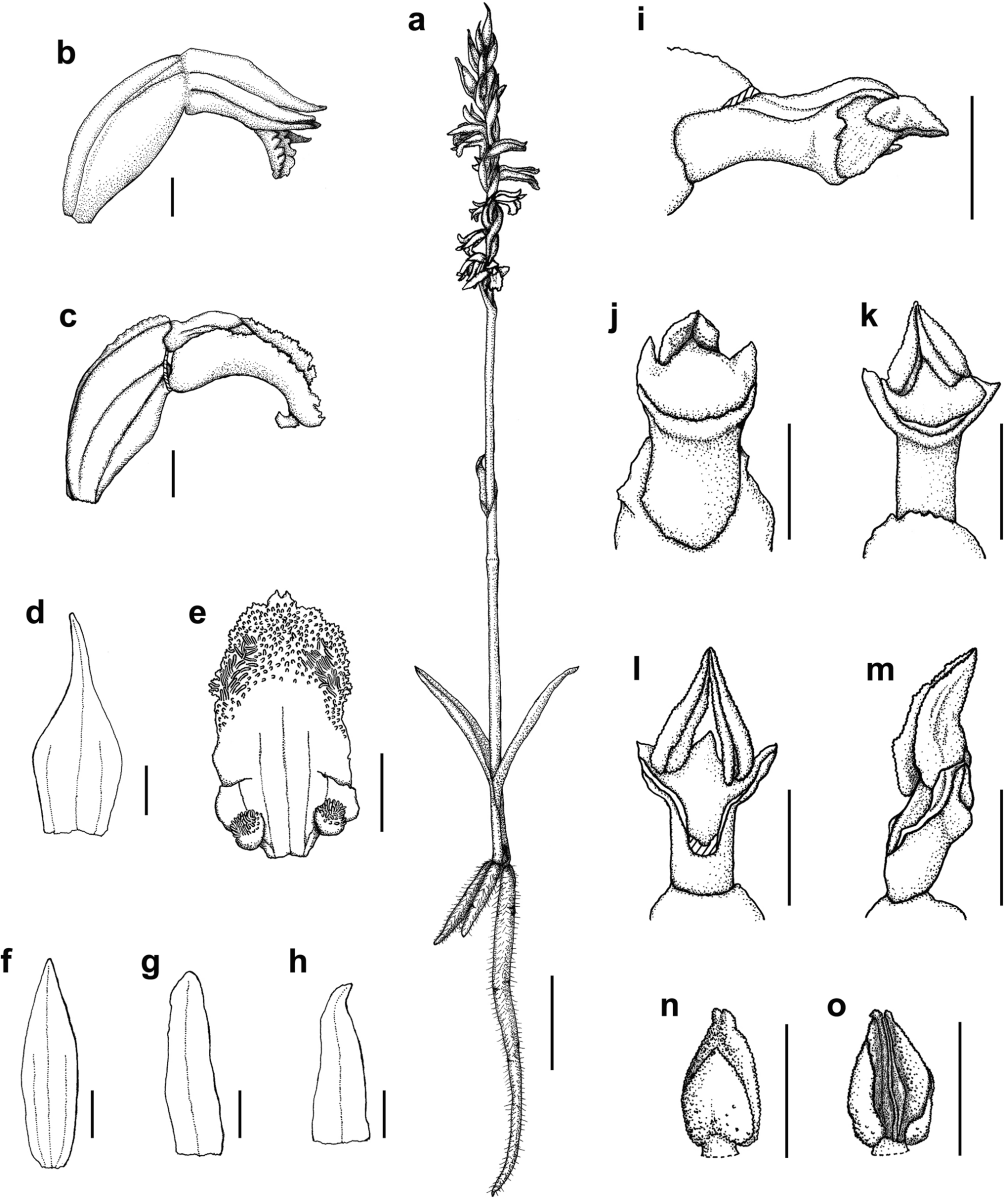
*Spiranthes hachijoensis*. **a** Habit. **b** Flower. **c** Column, labellum, and ovary. **d** Bract. **e** Flattened labellum. **f** Dorsal sepal. **g** Petal. **h** Lateral sepal. **i** Column, lateral view. **j**–**l** Column, bottom view. **m** Column, lateral view. **n**, **o** Anther cap. Scale bars: **a** 10 mm and **b**–**o** 1 mm. Drawn from *Katsushi Akita Ss152* (KYO; **a**–**h**, **k**, **n**–**o**), *Katsumi Iwahori KS246* (KYO; **i**–**j**), and *Emiko Kato KS244* (KYO; **l**, **m**) by Kumi Hamasaki

**Fig. 9:**
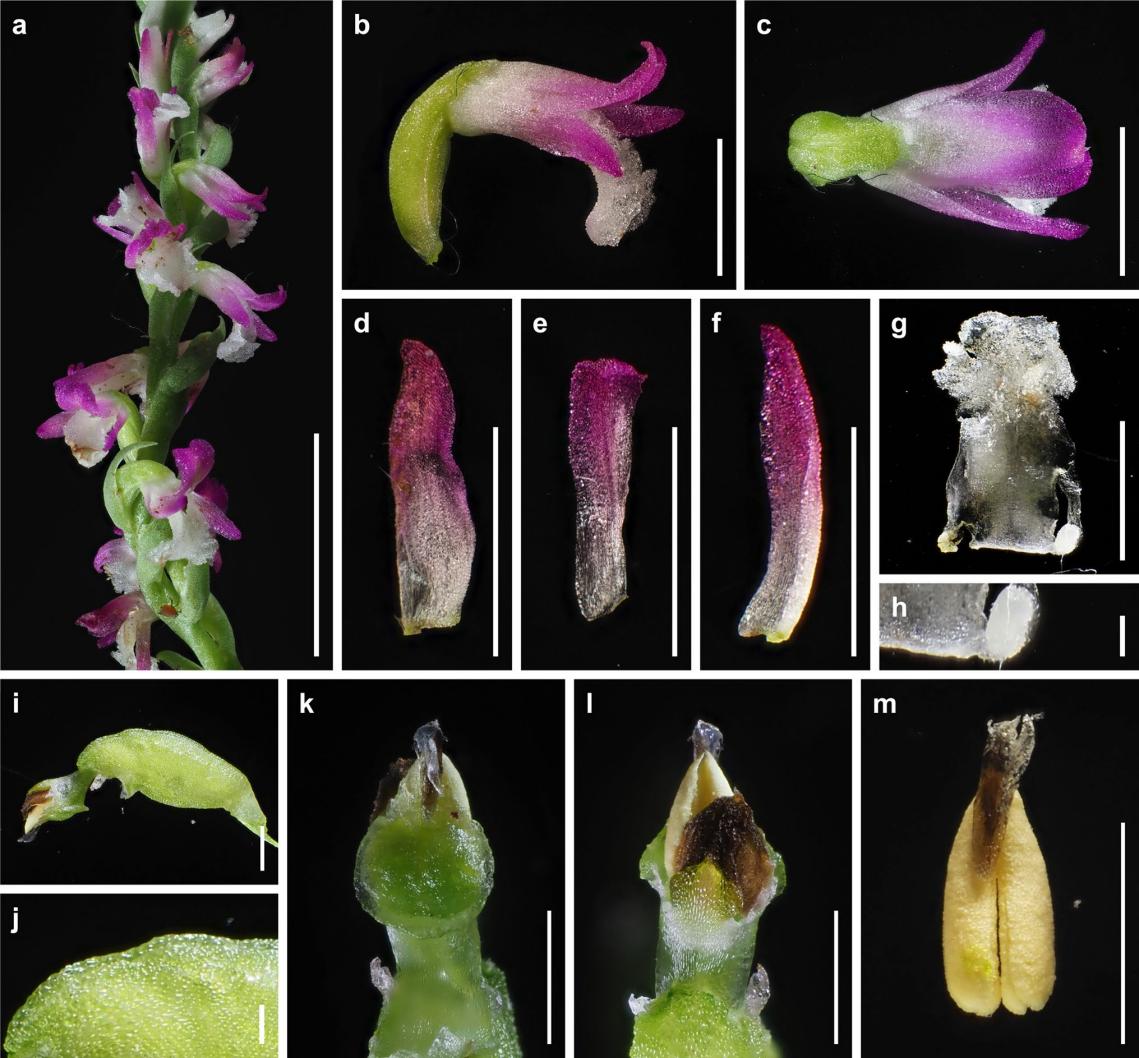
*Spiranthes sinensis* (*Masayuki Teruya Ss200*, KYO) on Okinawa Island, Okinawa Pref., Japan. **a** Inflorescence. **b** Flower, lateral view. **c** Flower, dorsal view. **d** Dorsal sepal. **e** Petal. **f** Lateral sepal. **g** Flattened labellum. **h** Close-up of basal callosities of labellum. **i** Ovary and column. **j** Close-up of glabrous ovary. **k** Column, bottom view. **l** Column, top view. **m** Pollinia with a viscidium. Scale bars: **a** 15 mm, **b**–**g** 3 mm, **h** 0.5 mm, **i**, **k**, **l** 1 mm, and **j** 0.3 mm. Photographed by Masayuki Ishibashi

The presence or absence of pubescence and viscidium and the shape of the stigma and labellum basal callosities have been considered consistent morphological characteristics for the delimitation of species within the *S. sinensis* species complex (Pace et al. 2019). *Spiranthes hachijoensis* is easily distinguishable from the sympatric *S. australis* by its papillate (vs. glabrous) basal labellum callosities and glabrous (vs. densely pubescent) rachis, ovaries, and sepals (Figs. 3, 4, 5, 6, 7, 8). Furthermore, *S. hachijoensis* can be distinguished from its putative parental species, *S. sinensis*, by its modified floral morphology associated with its autogamy. Specifically, *S. sinensis* exhibits a larger flower, a strongly recurved labellum that almost bends 360°, a suborbicular stigma, a well-developed rostellum that separates the stigma and pollinarium, and pollinia with a viscidium (Fig. 9). In contrast, *S. hachijoensis* has a smaller flower, a weakly recurved labellum that typically bends less than 270°, a crescent-shaped stigma, a degenerated rostellum, and pollinia without a viscidium (Figs. 3, 4, 5, 6, 7, 8). In addition to these morphological differences that result from distinct breeding systems, *S. hachijoensis* differs from *S. sinensis* by possessing globose (vs. conical to clavate) labellum basal callosities (Figs. 3, 4, 5, 6, 7, 8, 9). It is also noteworthy that the labellum basal callosities of *S. hachijoensis* are larger in proportion to the width of the base of the labellum compared to those of *S. sinensis* (Figs. 3, 4, 5, 6, 7, 8, 9).

Consequently, *S. hachijoensis* is morphologically similar to *S. hongkongensis* and *S*. *nivea* that are also autogamous (Lin and Lin 2011; Pace et al. 2019; Surveswaran et al. 2017). However, *S. hachijoensis* can be distinguished from *S. hongkongensis* by its glabrous (vs. densely pubescent) rachis, ovaries, and sepals. *Spiranthes hachijoensis* also differs from *S*. *nivea* in terms of its papillate (vs. nearly glabrous) labellum disc, larger papillate (vs. smaller glabrous) basal labellum callosities, and glabrous (vs. sparsely pubescent) rachis, ovaries, and sepals. *Spiranthes hachijoensis* appears to also be morphologically similar to *S. neocaledonica*, which has frequently been treated as a synonym of *S. sinensis* (Pace et al. 2019). Given that *S. neocaledonica* was described as having a short column and suppressed rostellum (Schlechter 1906), it is probably autogamous. Unfortunately, the type specimen of *S. neocaledonica* deposited at B (ZE Botanischer Garten und Botanisches Museum, Freie Universität Berlin) was destroyed during World War II, leaving insufficient reliable data to fully clarify the identity of this species. Nevertheless, according to the protologue (Schlechter 1906), *S. hachijoensis* differs from *S. neocaledonica* by having larger basal labellum callosities (0.5–0.7 mm long, versus 0.3 mm long). Moreover, it should be noted that the other autogamous lineages within the *S. sinensis* complex (i.e., *S. hongkongensis* and *S. nivea*) generally exhibit narrow geographical ranges (Pace et al. 2019; Suetsugu and Hsu 2023). Therefore, given the great geographical disjunction (*S. neocaledonica* in New Caledonia and *S. hachijoensis* in Japan) and the somewhat discrepant morphology, it is less likely that *S. hachijoensis* and *S. neocaledonia* belong to the same autogamous lineage. Overall, the molecular phylogeny reconstructed from MIG-seq data together with morphological and ecological analyses support the separation of *S. hachijoensis* as an independent species.

It is also noteworthy that the flower color of *S. hachijoensis* is highly polymorphic, varying from purple-pink to white (Figs. 3, 4, 5, 6, 7, 8), although species in the *S. sinensis* species complex often have pink flowers, and the members with entirely white flowers were traditionally recognized as a separate species only due to their white flowers (Pace and Cameron 2017). Similarly, even though bract length is a diagnostic character for certain *Spiranthes* species such as *S. nivea* (Lin and Lin 2011), *S. hachijoensis* exhibits tremendous variations in bract length (Figs. 3, 4, 5, 6, 7, 8). However, *S. hachijoensis* is a phylogenetically unified group with limited intraspecific genetic variation. Furthermore, the molecular phylogenetic analysis also confirmed that *S. nivea* var. *papillata* is a variant of *S. nivea*. However, it is morphologically distinguishable from *S. nivea* var. *nivea* by its more densely pubescent rachis and ovaries (vs. sparsely pubescent rachis and ovaries), narrower sepals with white tinged with pink or purple at apex (vs. wider and entirely white sepals), and papillose labellum disc (vs. almost glabrous labellum disc). These results indicate the need to reconsider the diagnostic characteristics of species delimitation in the *S. sinensis* species complex*.* As partly suggested by Pace et al. (2019), our molecular results suggested that floral color, bract length, and degree of pubescence should only be considered secondarily important features when investigating the systematics of the *S. sinensis* species complex, and that other characteristics, such as regional phenology, pubescence (presence/absence, not degree), and labellum and column morphology, should be given more emphasis. In particular, callosity papillae are likely to be a useful morphological feature that has been hitherto overlooked.

### Taxonomic treatment

***Spiranthes hachijoensis*** Suetsugu, *sp. nov.* (Figs. 3, 4, 5, 6, 7, 8).

**Type** JAPAN. Tokyo Metropolis: Hachijo Island, Okago, 15 May 2021, *Kyoko Kaneda & Mayumi Sugiura Ss205-1* (holotype: KYO).

**Diagnosis** *Spiranthes hachijoensis* is most morphologically similar to *S. hongkongensis* but can be distinguished by its glabrous rachis, ovaries, and sepals.

**Description** Plants 8–25 cm tall. Roots fleshy, fasciculate, slender to tuberous, up to ca. 5 mm in diameter. Leaves 2–6, basal, forming a rosette, erect and spreading, narrowly lanceolate, 19–85 × 4–9 mm, apex acute to acuminate, with indistinct petiole-like base. Inflorescence terminal, racemose erect, 8–25 cm, glabrous, with 1–3 sterile bracts sheathing peduncle; rachis 3.0–12.3 cm, with 16–50 spirally arranged flowers; floral bracts shorter or longer than pedicellate ovary, ovate-lanceolate, 3.2–15.2 mm long, 1.6–3.2 mm wide, glabrous, with acuminate apex. Ovary sessile or with inconspicuous pedicel, pale green, ellipsoid-obovoid, 3.7–6.7 mm long, 1.6–2.7 mm wide, glabrous. Flower resupinate, horizontal or nodding, weakly opening. Dorsal sepal entirely white or pinkish purple with a basal white part, narrowly triangular, 3.2–4.3 mm long, 0.9–1.6 mm wide, glabrous, apex obtuse or rarely acute, connivent with petals and forming hoods over column. Lateral sepals entirely white or pinkish purple with a basal white part, narrowly triangular to lanceolate, slightly oblique approximately halfway along length, 3.7–4.2 mm long, 0.7–1.6 mm wide, glabrous, apex obtuse or acute. Petals entirely white or pinkish purple with a basal white part, linear to oblanceolate, occasionally slightly oblique approximately halfway along length, 3.1–3.5 mm long, 0.7–0.9 mm wide, glabrous, apex rounded. Labellum entirely white sometimes tinged with pink or purple at apex, recurved downward approximately two-thirds from claw to labellum apex, oblong to slightly constricted near reflection and then dilating below, centrally papillate near apex, 3.2–3.5 mm long, 1.4–1.8 mm wide below callosities, 1.4–1.9 mm wide at widest point below recurvature; margin entire to slightly undulating from base until area of recurvature, below point of recurvature margin becoming ruffled and lacerate; two basal callosities transparent, globose, papillate, 0.5–0.7 mm long, 0.5–0.8 mm wide. Column translucently white to pale green dorsally, pale green ventrally, clavate, 1.3–2.0 mm long; anther cap yellow–brown, ovate, partly embedded on upper part of column, 0.9–1.1 mm long; pollinia 2, each 2-partite, yellow, granular-farinaceous, without viscidium at narrower end, 0.9–1.1 mm long; rostellum narrow, typically lacking; stigma crescent-shaped, distinctly trilobed filled with viscid liquid, 0.6–1.3 mm long. Fruit ellipsoid-obovoid, 4.3–7.7 mm long, 2.0–3.0 mm wide, glabrous. Seed fusiform, ca. 0.6 mm long; embryo 1, ellipsoid, ca. 0.15 mm long.

**Additional specimens examined (paratype)** JAPAN. **Kagoshima Pref.**: Kagoshima-shi, Kamoikeshinmachi, 25 April 2021, *Nahoko Fukudome Ss203-1* (KYO, spirit collection); loc. cit., 25 April 2021, *Nahoko Fukudome Ss203-2* (KYO); loc. cit., 25 April 2021, *Nahoko Fukudome Ss203-3* (KYO, spirit collection); Kagoshima-shi, Honkoshinmachi, 09 May 2021, *Hibiki Katayama KS869* (KYO). **Miyazaki Pref.**: Nichinan-shi, 20 May 2017, *Kenji Suetsugu KS231* (TNS); loc. cit., 20 May 2017, *Kenji Suetsugu HH660* (SPMN). **Kochi Pref.**: Konan-shi, Noichi-cho, 13 May 2022, *Shohei Fujimori F220513-1* (TNS). **Gifu Pref.**: Ena-shi, Oi-cho, 6 May 2018, *Katsumi Iwahori KS246* (TNS); Mino-shi, Izumi-cho, 3 June 2016, *Ayako Yoshii 2* (TNS). **Aichi Pref.**: Nagoya-shi, Midori-ku, Sakaimatsu, 18 May 2019, *Noriyuki Sasaki KS396* (TNS); Nagoya-shi, Minami-ku, Daido-cho, 28 May 2021, *Shohei Ohe Ss213* (KYO); Nagoya-shi, Minami-ku, Motohoshizaki-cho, 29 May 2021, *Shohei Ohe Ss214* (KYO); Kariya-shi, Hitotsugi-cho, 27 May 2021, *Tetsuya Okazaki Ss215* (KYO). **Tokyo Metropolis**: Hachijo Island, Okago, 24 May 2017, *Yumiko Ohba HH660* (SPMN); loc. cit., 24 May 2017, *Yumiko Ohba HH661* (SPMN); loc. cit., 15 May 2021, *Kyoko Kaneda & Mayumi Sugiura Ss205-1* (KYO); loc. cit., 22 May 2021, *Kyoko Kaneda & Mayumi Sugiura Ss205-4* (KYO); loc. cit., 11 May 2020, *Kyoko Kaneda, Mayumi Sugiura Ss205-9* (KYO); loc. cit., 11 June 2005, *Hiroko Nakayama KPM-NA0126192* (KPM); Hachijo Island, Mitsune, 24 May 2017, *Yumiko Ohba HH665* (SPMN); loc. cit., 24 May 2017, *Yumiko Ohba Ss122* (SPMN); loc. cit., 24 May 2017, *Yumiko Ohba Ss123* (SPMN); loc. cit., 24 May 2017, *Yumiko Ohba Ss124* (SPMN); loc. cit., 25 May 2018, *Katsushi Akita KS275* (KYO); Shinagawa-ku, Koyama, 12 May 2021, *Akari Yoshida Ss211* (KYO). **Chiba Pref.**: Ichihara-shi, Okubo, 28 June 2015, *Emiko Kato HH107* (SPMN); loc. cit., 12 May 2016, *Kenji Suetsugu Ss17* (KYO); loc. cit., 15 May 2018, *Emiko Kato KS244* (TNS); Ichihara-shi, 12 May 2005, *Emiko Kato CBM-BS-234363* (CBM); Kimitsu-shi, Toyofusa, 28 June 2015, *Emiko Kato HH106* (SPMN); loc. cit., 12 May 2016, *Kenji Suetsugu Ss18* (KYO); loc. cit., 12 May 2016, *Kenji Suetsugu Ss19* (KYO, spirit collection); Kimitsu-shi, Kawayatsu 12 May 2016, *Kenji Suetsugu Ss14* (KYO); loc. cit. 12 May 2016, *Kenji Suetsugu Ss15* (KYO, spirit collection). **Ibaraki Pref.**: Tsukuba-shi, Amakubo, 31 May 2015, *Shohei Fujimori F150531-1* (TNS); Tsukuba-shi, Oda, 24 June 2014, *Shohei Fujimori F140624-1* (TNS); 10 June 2015, *Shohei Fujimori F150610-1* (TNS).

**Japanese name** Hachijo-neji-bana.

**Distribution** Japan (Kyushu District [Kagoshima and Miyazaki], Shikoku District [Kochi], Chubu District [Aichi, and Gifu], and Kanto District [Tokyo, Chiba, and Ibaraki]). Notably, *S. hachijoensis* has a more northerly distribution than its putative outcrossing ancestral species *S. sinensis* (Maekawa 1971; Satomi 1982; Suetsugu and Hayakawa 2019).

**Conservation status** While we found that *S. hachijoensis* is distributed in Kyushu, Shikoku, Chubu, and Kanto districts, *S. hachijoensis* is much rarer than its sympatric closely related species *S. australis.* Each population often harbors less than 20 individuals, and even in the type locality that sustains the largest number of individuals, the population size is approximately 100. Therefore, we classify its conservation status as "vulnerable" (VU) based on IUCN criteria (IUCN 2019) under criterion D1, which states that the number of mature individuals is less than 1000.

## Supplementary Information

Below is the link to the electronic supplementary material.

Supplementary file1 (XLSX 20 KB)

## Data Availability

MIG-seq data are deposited at the NCBI Sequence Read Archive (accession number: PRJNA907989).
